# Educational and behavioural interventions for reducing fear of hypoglycaemia in adults with type 2 diabetes: A systematic review and meta-analysis of randomised controlled trials

**DOI:** 10.1371/journal.pone.0358881

**Published:** 2026-10-05

**Authors:** Yan Tang, Junjun Zhou, Wan Ying Gan, Kim Geok Soh, Kim Lam Soh

**Affiliations:** 1 Department of Nursing, Universiti Putra Malaysia, Serdang, Selangor, Malaysia; 2 Faculty of Nursing, Jiangsu Medical College, Yancheng, Jiangsu, China; 3 Department of Endocrinology, Yancheng No.1 People’s Hospital, Affiliated Hospital of Medical School, Nanjing University, Yancheng, Jiangsu, China; 4 Department of Nutrition, Universiti Putra Malaysia, Serdang, Selangor, Malaysia; 5 Department of Sports Studies, Universiti Putra Malaysia, Serdang, Selangor, Malaysia; Shuguang Hospital, CHINA

## Abstract

**Background:**

Fear of hypoglycaemia (FoH) can interfere with diabetes self-management in adults with type 2 diabetes mellitus (T2DM). Educational and behavioural interventions may address hypoglycaemia-related worry and avoidance, but evidence focused on adults with T2DM remains limited. This systematic review and meta-analysis examined the effects of structured educational and behavioural interventions on FoH in this population.

**Methods:**

Nine databases were searched from inception to 31 December 2025 for randomised controlled trials of educational and behavioural interventions addressing FoH or FoH-related outcomes in adults with T2DM. Data were pooled using random-effects models, estimating standardised mean differences (SMDs) with 95% confidence intervals (CIs). Heterogeneity was assessed using Tau² and I², and a 95% prediction interval was estimated. Small-study effects and additional sensitivity analyses were examined. Risk of bias was assessed using Cochrane RoB 2, and certainty of evidence was assessed using GRADE. The review was registered with PROSPERO (CRD42025630957).

**Results:**

Eleven RCTs involving 1,096 participants were included. The exploratory pooled average effect favoured educational and behavioural interventions for reducing FoH (SMD = −1.89; 95% CI −2.54 to −1.25; *p* < 0.00001). Heterogeneity was substantial (Tau² = 1.10; I² = 95%), and the 95% prediction interval crossed the line of no effect (95% PI −4.34 to 0.56), suggesting that effects may differ across future similar studies. Funnel plot asymmetry and Egger’s regression test suggested possible small-study effects. Trim-and-fill findings were estimator-dependent and suggested possible attenuation of the pooled estimate in exploratory analysis. The certainty of evidence was very low because of risk of bias, substantial heterogeneity, indirectness, and possible small-study effects.

**Conclusion:**

Very low-certainty evidence suggests that structured educational and behavioural interventions may reduce FoH in adults with T2DM; however, the magnitude and generalisability of this effect remain uncertain. Further RCTs with standardised FoH measures, longer follow-up, and clearer reporting of intervention components and fidelity are needed.

## 1. Introduction

The global health and economic burden of diabetes continues to increase, affecting both individuals and healthcare systems [1]. Type 2 diabetes mellitus (T2DM), characterised by insulin resistance and β-cell dysfunction, accounts for approximately 90–95% of all diabetes cases and is associated with persistent hyperglycaemia and long-term complications if inadequately managed [2,3].

Although glycaemic control is central to diabetes management, intensive glucose-lowering treatment—particularly regimens involving insulin or insulin secretagogues—can increase the risk of hypoglycaemia [4], a common but potentially serious acute complication [5]. Recurrent hypoglycaemia may undermine treatment adherence and reduce quality of life [6]. It can also lead to excessive concern and behavioural avoidance, which are core features of fear of hypoglycaemia (FoH) [7]. FoH involves both emotional responses, such as anxiety and worry, and maladaptive behaviours, such as defensive overeating or arbitrarily reducing insulin doses, intended to avoid future episodes [8]. These responses may in turn compromise self-management and glycaemic control, creating a cycle of suboptimal metabolic regulation and psychological distress [7,9].

Despite increasing recognition of its clinical importance, FoH remains under-recognised and insufficiently addressed among adults with T2DM [10,11]. Existing evidence suggests that older age, greater comorbidity burden, impaired awareness of hypoglycaemia, and insulin therapy may increase the risk of FoH in this population [12–14]. Although a range of interventions has been developed, from technology-based approaches to cognitive-behavioural and psychosocial support [15], most intervention research has focused on people with type 1 diabetes (T1DM), in whom FoH has often been assessed as a secondary outcome [16,17]. To our knowledge, no previous systematic review and meta-analysis has specifically evaluated educational and behavioural interventions targeting FoH exclusively in adults with T2DM.

Given the distinct clinical and experiential context of FoH in T2DM, tailored intervention strategies are needed [10]. Therefore, this systematic review and meta-analysis was conducted to evaluate the available evidence on structured educational and behavioural interventions for reducing FoH among adults with T2DM. By synthesising the available evidence, this study aims to clarify the current evidence base and identify methodological and clinical considerations for future intervention development in this population.

## 2. Methods

The protocol for this review was prospectively registered with PROSPERO (CRD42025630957) on 16 January 2025 and is available from the PROSPERO database.

The review followed the Cochrane Handbook for Systematic Reviews of Interventions, version 6.3 [18] and was reported in accordance with the PRISMA 2020 statement [19].

### 2.1 Search strategy

Nine databases were searched: Cochrane Library, Scopus, PubMed, Web of Science, Embase, PsycINFO, CINAHL, WANFANG, and CNKI. Searches covered the period from database inception to 31 December 2025. The search strategy combined Medical Subject Headings (MeSH) and free-text terms for type 2 diabetes mellitus, hypoglycaemia-related fear or worry, and educational or behavioural interventions, using Boolean operators. Only randomised controlled trials (RCTs) were considered eligible. A bridge search was conducted on 20 April 2026 to identify newly published eligible trials. No additional eligible trial meeting the inclusion criteria was identified. The full search strategy is provided in S1 File.

### 2.2 Eligibility criteria

Eligibility criteria were defined using the PICOS framework.

#### Participants.

Studies were eligible if they included adults aged 18 years or older with type 2 diabetes mellitus (T2DM) and reported fear of hypoglycaemia (FoH), hypoglycaemia-related worry, or hypoglycaemia-related avoidance measured with a validated FoH-related instrument. Studies focusing exclusively on type 1 diabetes, gestational diabetes, secondary diabetes, children, or caregivers were excluded unless separate adult T2DM data were available.

#### Interventions.

Eligible interventions were structured educational and/or behavioural strategies intended to reduce FoH, hypoglycaemia-related worry, or hypoglycaemia-related avoidance. These included psychological therapies, diabetes self-management education, behaviour modification, family-based support, nursing-led education, and digitally supported interventions delivered in clinical, community, or digital settings.

#### Comparisons.

Eligible comparators included usual care, standard education, minimal intervention, wait-list control, placebo control, or active control interventions that did not specifically target FoH.

#### Outcomes.

The primary outcome was FoH measured using validated psychometric scales or FoH-related subscales. Studies reporting only glycaemic outcomes without a FoH-related psychological outcome were excluded.

#### Study design.

Only RCTs were eligible. Quasi-experimental studies, observational studies, qualitative studies, case reports, reviews, editorials, and conference abstracts with incomplete data were excluded. Peer-reviewed articles published in English or Chinese were included.

### 2.3 Study selection and screening

All retrieved records were imported into a reference management database, and duplicates were removed. Two reviewers independently screened titles and abstracts against the eligibility criteria. The same reviewers then independently assessed the full texts of potentially eligible articles. Disagreements were resolved through discussion or, when necessary, consultation with a third reviewer.

### 2.4 Data extraction

A standardised data extraction form was developed and applied independently by two reviewers. Disagreements were resolved by discussion or, when required, adjudication by a third reviewer. Extracted information included first author, publication year, country, study design, sample size, participant characteristics, FoH eligibility criteria, intervention type, intervention duration, comparator, assessment time points, FoH measurement tools, and outcome data.

For the main meta-analysis, one FoH effect estimate was selected from each trial to avoid double counting. The selected estimate corresponded to the main intervention endpoint or the primary post-intervention/follow-up assessment considered most relevant to the intervention effect. When both total FoH scores and subscale scores were reported, total FoH scores were prioritised. When total scores were unavailable, the FoH worry subscale or the most directly FoH-related validated scale component was used. Additional later follow-up data from the same study were not included again in the main analysis but were used only in exploratory subgroup analyses by follow-up duration, where applicable. All FoH scales were harmonised so that higher scores indicated greater FoH; therefore, negative SMDs favoured the intervention group. When standard errors (SEs) rather than standard deviations (SDs) were reported, SDs were calculated as SD = SE × √n, where n represented the relevant group sample size. The dataset used for the meta-analysis is provided in S2 File.

For studies with multiple eligible intervention arms, the intervention arm most directly aligned with the review question was selected to avoid double counting. Studies published in English or Chinese were eligible for inclusion. Where clarification of reported data was required, extraction was verified using the published article, supplementary material, or author correspondence, where available.

### 2.5 Risk of bias and certainty of evidence assessment

Risk of bias in the included RCTs was assessed independently by two reviewers using the revised Cochrane Risk of Bias tool for randomised trials (RoB 2) [20]. The assessment covered bias arising from the randomisation process, deviations from intended interventions, missing outcome data, measurement of the outcome, and selection of the reported result. Each domain and the overall risk of bias were rated as low risk, some concerns, or high risk. Disagreements were resolved through discussion or adjudication by a third reviewer.

Particular attention was given to bias in measurement of the outcome because FoH was assessed using self-reported instruments. Blinding of participants was generally not feasible in educational, behavioural, psychological, nursing-led, family-based, or digitally supported interventions; therefore, participants were likely to be aware of their allocation. For the main FoH outcome, this domain was rated as some concerns where knowledge of allocation could plausibly have influenced self-reported FoH scores.

The certainty of evidence for the primary outcome was assessed using the Grading of Recommendations Assessment, Development and Evaluation (GRADE) approach. As the included studies were RCTs, the initial certainty rating was high and was downgraded where appropriate for risk of bias, inconsistency, indirectness, imprecision, and publication bias. The GRADE assessment considered RoB 2 judgements for risk of bias, heterogeneity and prediction interval findings for inconsistency, geographic concentration and intervention/contextual heterogeneity for indirectness, precision of the pooled effect estimate for imprecision, and funnel plot asymmetry, Egger’s regression test, and trim-and-fill findings for publication bias.

### 2.6 Data synthesis and analysis

Meta-analysis was performed when sufficient data were available for FoH outcomes. Because the included trials used different validated FoH instruments or FoH-related subscales, standardised mean differences (SMDs) with 95% confidence intervals (CIs) were calculated [21]. Analyses were conducted using Review Manager (RevMan), version 5.4 [22]. A random-effects inverse-variance model was specified a priori because clinical and methodological heterogeneity was expected across intervention content, delivery format, follow-up duration, participant characteristics, and FoH measurement tools. Between-study variance (Tau²) was estimated using the DerSimonian–Laird method, as implemented in RevMan 5.4.

Statistical heterogeneity was assessed using the Chi² test, Tau², and the I² statistic. An I² value greater than 50% was considered to indicate substantial heterogeneity [23]. Given the expected heterogeneity, a 95% prediction interval was calculated using the pooled SMD, its standard error, Tau², and the t distribution with k − 1 degrees of freedom, where k represented the number of studies included in the main meta-analysis. The prediction interval was used to estimate the range of true effects that might be expected in future similar studies. When heterogeneity was substantial, the pooled estimate was interpreted as an exploratory average effect rather than as an estimate of a common intervention effect.

Subgroup analyses were conducted by follow-up duration (≤3 months vs. > 3 months) and intervention category (nursing/education-based vs. psychological/behavioural theory-based). Where a trial contributed more than one eligible time point to a subgroup analysis, the subgroup findings were treated as exploratory and interpreted cautiously. A leave-one-out sensitivity analysis was performed to assess whether the pooled effect was influenced by any single study.

Publication bias and small-study effects were examined by visual inspection of funnel plots and Egger’s regression test when at least 10 studies were available [24]. Duval and Tweedie trim-and-fill analyses were additionally performed as exploratory assessments of the potential influence of small-study effects on the pooled estimate.

Post hoc sensitivity analyses were conducted to examine the robustness, contextual concentration, and generalisability of the findings. These included a sensitivity analysis excluding studies published in Chinese-language domestic journals with limited international visibility and exploratory geographic sensitivity analyses comparing China-based and non-China studies. These post hoc analyses were interpreted cautiously and were used to examine robustness rather than to establish comparative intervention effects.

### 2.7 Ethics statement

This study was a systematic review and meta-analysis of previously published studies. No new human participants, human specimens, animals, or field materials were recruited or collected by the authors; therefore, ethical approval and informed consent were not required.

## 3. Results

### 3.1 Study selection

The database search identified 3,118 records. After duplicates were removed, 1,862 unique records were screened by title and abstract. Ninety-two full-text articles were assessed for eligibility, and 11 studies met the inclusion criteria. All included studies were randomised controlled trials (RCTs) and reported FoH-related outcomes suitable for synthesis. Fig 1 presents the PRISMA 2020 flow diagram for study selection.

**Fig 1:**
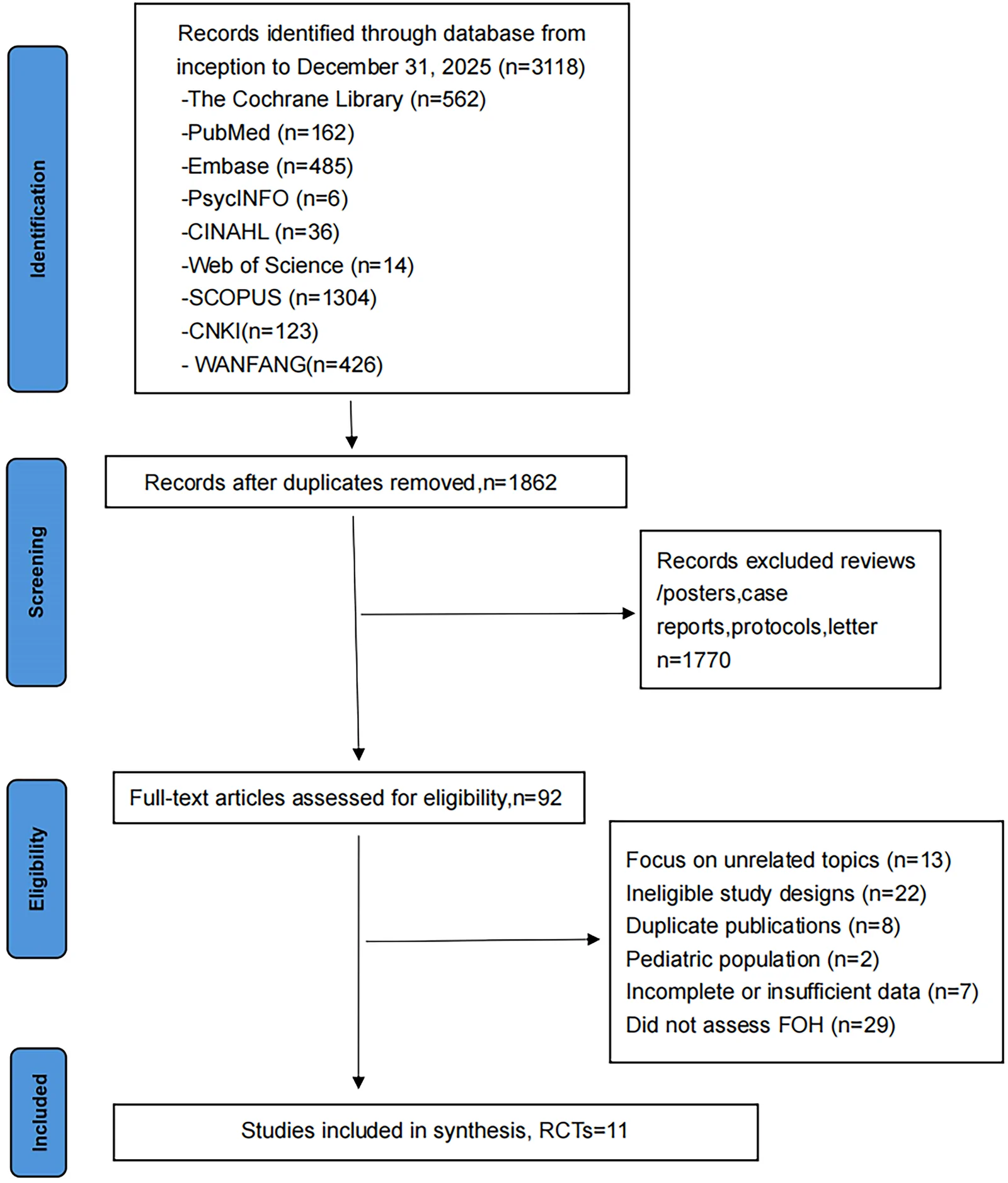
PRISMA flow diagram for study selection. The diagram shows the number of records identified, screened, assessed for eligibility, and included in the review, with reasons for full-text exclusions. Database searches covered the period from inception to 31 December 2025, and a bridge search was conducted on 20 April 2026.

### 3.2 Study characteristics

The 11 included RCTs were published between 2017 and 2025. Their key characteristics are summarised in Table 1. The studies were conducted in four countries: China (n = 8) [25–32], Indonesia (n = 1) [33], Iran (n = 1) [34], and Turkey (n = 1) [35]. Sample sizes ranged from 50 to 228 participants, with 1,096 participants included across all trials. The mean age of participants ranged from approximately 41–71 years.

**Table 1:** Characteristics of included studies.

Study	Country	Design/ sample size	Participants and FoH criteria	Mean age/ diabetes duration	Intervention and duration	Assessment timepoints	Outcomes assessed	FoH tool/ FoH finding
Yan et al. 2024 [25]	China	RCT; n = 60/60	Newly diagnosed T2DM patients	Age: I 47.88, C 45.17; diabetes duration: not reported	Online feedback intervention based on King’s Theory; 24 weeks	Baseline and 24 weeks	Glycaemic indices, FoH, alexithymia	CHFS-II; reduction in behaviour and worry scores reported
Shen et al. 2022 [26]	China	RCT; n = 39/39	T2DM with impaired awareness of hypoglycaemia, defined by Gold score ≥4	Age: I 57.97, C 57.82; duration: I 9.41, C 10.92 years	CSM-based illness perception education with follow-up; 12 weeks	Baseline, 1 month, and 3 months	Illness perceptions, coping styles, FoH, hypoglycaemia awareness	CHFS-II-WS; small reduction in extracted final FoH score
Sheikhi et al. 2020 [34]	Iran	RCT; n = 36/36	T2DM patients with FoH and hypoglycaemic symptoms	Age: I 43.17 ± 10.55, C 45.86 ± 13.60; duration: I 10.5, C 10.4 years	EMDR psychological therapy; two 45-min sessions	Baseline, 1 month, and 3 months	FoH, hypoglycaemia severity, demographic variables	HFS-II; reduction in FoH score reported
Xu et al. 2023 [27]	China	RCT; n = 114/114	T2DM inpatients with FoH; FoH eligibility not assessed using a standardised scale	Age: I 70.91 ± 2.85, C 70.95 ± 2.87; duration: not reported	ESCA and IMB model-based targeted nursing intervention; during hospitalisation	Before and after intervention	Hypoglycaemia incidence, ESCA, FoH	CHFS-II-WS; reduction in FoH score reported
Liu et al. 2023 [29]	China	RCT; n = 45/45	Patients with T2DM who had experienced hypoglycaemia	Age: I 57.47 ± 5.78, C 58.62 ± 6.14; duration: I 10.04 ± 2.46, C 10.96 ± 2.08 years	Hope theory-based education and psychological support; 4 weeks	Before and after intervention	Blood glucose, FoH	HFS-II; reduction in FoH score reported
Liang et al. 2021 [28]	China	RCT; n = 50/50	T2DM patients with CHFS-II-WS score >25	Age: I 41.09 ± 12.87, C 42.12 ± 12.57; duration: ≥ 1 year	Stress inoculation training programme; 8 weeks	Before and after intervention	FoH, empowerment, self-management behaviour	CHFS-II-WS; reduction in FoH score reported
Zhou et al. 2019 [30]	China	RCT; n = 60/60	Patients with T2DM who had experienced hypoglycaemia	Age: I 63.2 ± 5.9, C 62.8 ± 6.3; duration: I 6.5 ± 1.1, C 6.1 ± 1.4 years	Multidisciplinary in-hospital consultation and education; during hospitalisation	Before intervention and 6 months after intervention	FoH, blood glucose, quality of life	HFS-II-WS; reduction in FoH score reported
Lin et al. 2017 [31]	China	RCT; n = 43/43	Patients with T2DM who had experienced hypoglycaemia	Age: I 63.01 ± 4.57, C 62.68 ± 4.35; duration: I 5.96 ± 1.34, C 6.03 ± 1.29 years	Traditional Chinese medicine-based emotional nursing; during hospitalisation	Before and at the end of intervention	FoH, blood glucose	HFS-II-WS; reduction in FoH score reported
Zhao et al. 2025 [32]	China	Pilot RCT; n = 25/25	T2DM with elevated FoH, defined by HFS-WS item score ≥3	Age: I 60.6, C 59.8; duration: I 10.2, C 10.8 years	BCW-based health education with in-person sessions and online coaching; inpatient phase plus 3 weeks online coaching	T0, T1, and T2	FoH behaviour and worry, impaired hypoglycaemia awareness, medical support, self-management attitudes	HFS-BS, HFS-WS, HFS total; reduction in FoH behaviour, worry, and total scores reported
Büyükkaya Besen et al. 2025 [35]	Turkey	RCT; n = 35/32	T2DM newly initiated on insulin therapy	Age: I 54.57 ± 9.79, C 54.84 ± 7.74; duration: I 7.09 ± 4.16, C 6.84 ± 4.19 years	Digital diabetes clinic continuous support programme; 12 weeks active support	Baseline, 3 months, and 6 months	HbA1c, LDL, triglycerides, hypoglycaemia frequency, insulin management, emergency visits, treatment adherence	HFS; reduction in hypoglycaemia fear reported
Rondhianto et al. 2024 [33]	Indonesia	Randomized controlled pretest-posttest design; n = 41/44	T2DM with recent hypoglycaemia; family-caregiver dyads	Age: 48.6 ± 10.2; duration: 4.7 ± 3.2 years	Family Caregiver Empowerment Model; 10 weeks	Pre-test and post-test 2 weeks after intervention	FoH	FH-15; lower final FoH score in the intervention group compared with control

*Note:* BCW, Behavior Change Wheel; CHFS-II, Chinese version of the Hypoglycaemia Fear Survey-II; CHFS-II-WS, Chinese version of the Hypoglycaemia Fear Survey-II Worry Scale; CSM, Common-Sense Model; EMDR, Eye Movement Desensitisation and Reprocessing; ESCA, Exercise of Self-Care Agency Scale; FH-15, Fear of Hypoglycaemia Scale-15; FoH, fear of hypoglycaemia; HFS, Hypoglycaemia Fear Survey; HFS-BS, Hypoglycaemia Fear Survey Behaviour Scale; HFS-II, Hypoglycaemia Fear Survey-II; HFS-WS, Hypoglycaemia Fear Survey Worry Scale; IMB, Information-Motivation-Behavioural Skills; I, intervention group; C, control group; RCT, randomised controlled trial; T2DM, type 2 diabetes mellitus.

The study populations varied in clinical characteristics and FoH-related eligibility criteria. One study included individuals with T2DM newly initiated on insulin therapy [35], one included newly diagnosed T2DM patients [25], and one recruited participants with impaired awareness of hypoglycaemia [26]. The remaining studies mainly included adults with T2DM who had experienced hypoglycaemia or had elevated FoH at baseline [27–34]. FoH was measured using validated instruments or validated FoH-related subscales, including HFS-II, HFS-II-WS, HFS-BS, CHFS-II, CHFS-II-WS, and FH-15.

### 3.3 Risk of bias assessment

Risk of bias was assessed using the RoB 2 tool and is summarised in Fig 2, with domain-level judgements and supporting reasons provided in S3 File. All 11 included trials were judged as raising some concerns overall, and no study was judged to be at high risk of bias. A common reason for some concerns was the measurement of the outcome, because FoH was assessed using self-reported instruments in trials where blinding of participants was generally not feasible. Knowledge of intervention allocation could therefore plausibly have influenced self-reported FoH scores.

**Fig 2:**
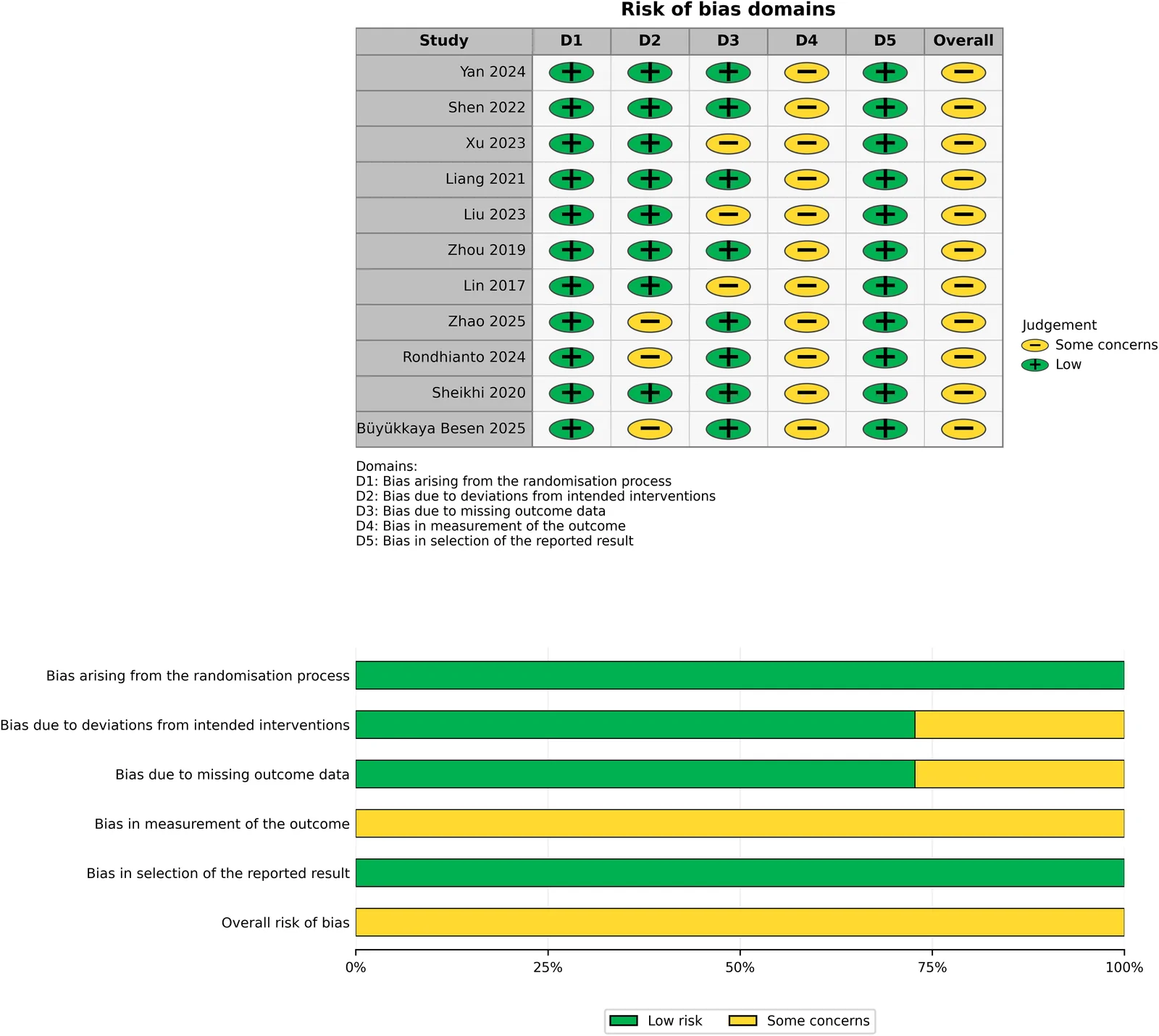
Risk of bias assessment for the included randomised controlled trials. Risk of bias was assessed using the Cochrane RoB 2 tool across five domains. Green symbols indicate low risk, and yellow symbols indicate some concerns. No included study was judged to be at high risk of bias.

Some studies also raised concerns in other domains, including deviations from intended interventions or missing outcome data, depending on the completeness of intervention adherence, follow-up, and reporting. However, no study showed sufficient concerns across domains to warrant an overall high-risk judgement. These RoB 2 judgements were incorporated into the GRADE assessment.

### 3.4 Narrative synthesis of intervention characteristics and outcomes

#### 3.4.1 Intervention characteristics and classification.

The interventions varied in theoretical basis, content, duration, delivery mode, and intensity. Based on their main theoretical or implementation approach, interventions were grouped into two broad categories: nursing theory or education-based interventions (n = 7) [25,27,29–31,33,35] and psychological or behavioural theory-based interventions (n = 4) [26,28,32,34]. Most control groups received routine diabetes care, standard education, or usual follow-up.

Programme duration ranged from brief or inpatient interventions to longer programmes lasting up to 24 weeks [25]. Several interventions were delivered over four to twelve weeks [26,28,29,32,33,35]. Delivery formats included face-to-face education, psychological therapy, inpatient nursing education, family caregiver empowerment, WeChat- or telephone-based follow-up, and digital clinic support. Intervention providers included nurses, psychologists, diabetes educators, and multidisciplinary teams.

A TIDieR-style summary of intervention characteristics is provided in S1 Table.

#### 3.4.2 FoH-related outcomes.

All 11 included studies assessed FoH or FoH-related worry and avoidance. In several trials, FoH was the primary or central outcome; in others, it was assessed alongside glycaemic, behavioural, or psychosocial outcomes. FoH was measured using HFS-related instruments or subscales [29–32,34,35], Chinese versions of HFS-related measures [25–28], or FH-15 [33].

Most studies reported lower FoH scores in the intervention group after the intervention or at follow-up [25,27–35]. The direction of effects generally favoured educational and behavioural interventions, although the magnitude varied across studies. One study using a Common-Sense Model-based illness perception intervention showed only a small effect on the extracted FoH final score [26]. Overall, the narrative findings were broadly aligned with the quantitative synthesis, but variability in population characteristics, intervention content, measurement tools, and effect sizes supported cautious interpretation of the pooled estimate.

#### 3.4.3 Glycaemic and psychosocial outcomes.

Several studies also reported changes in glycaemic or psychosocial outcomes. Improvements in HbA1c, fasting blood glucose, or 2-hour postprandial blood glucose were reported in some trials [25,29–31,35]. Other reported outcomes included self-efficacy, confidence in insulin management, illness perceptions, coping style, empowerment, family caregiver support, and quality of life [26,28,30,33,35]. These outcomes were not pooled in the present meta-analysis because the measures and reporting formats varied across studies.

#### 3.4.4 Variation in outcome measurement and intervention effects.

The included studies differed in intervention duration, delivery setting, intensity, and follow-up length. FoH was assessed using different validated instruments or subscales, including HFS-II, HFS-II-WS, HFS-BS, CHFS-II, CHFS-II-WS, and FH-15. These differences supported the use of SMDs for quantitative synthesis, but they may also have contributed to between-study heterogeneity. Although the direction of effect was generally in favour of the intervention groups, the size of the effect varied substantially across studies.

### 3.5 Meta-analysis of FoH outcomes

#### 3.5.1 Overall effect on FoH.

Eleven RCTs involving 1,096 participants reported FoH outcomes using validated instruments or validated FoH-related subscales [25–35]. A random-effects meta-analysis showed that the exploratory pooled average effect favoured educational and behavioural interventions compared with control conditions (SMD = −1.89; 95% CI −2.54 to −1.25; *p* < 0.00001) (Fig 3).

**Fig 3:**
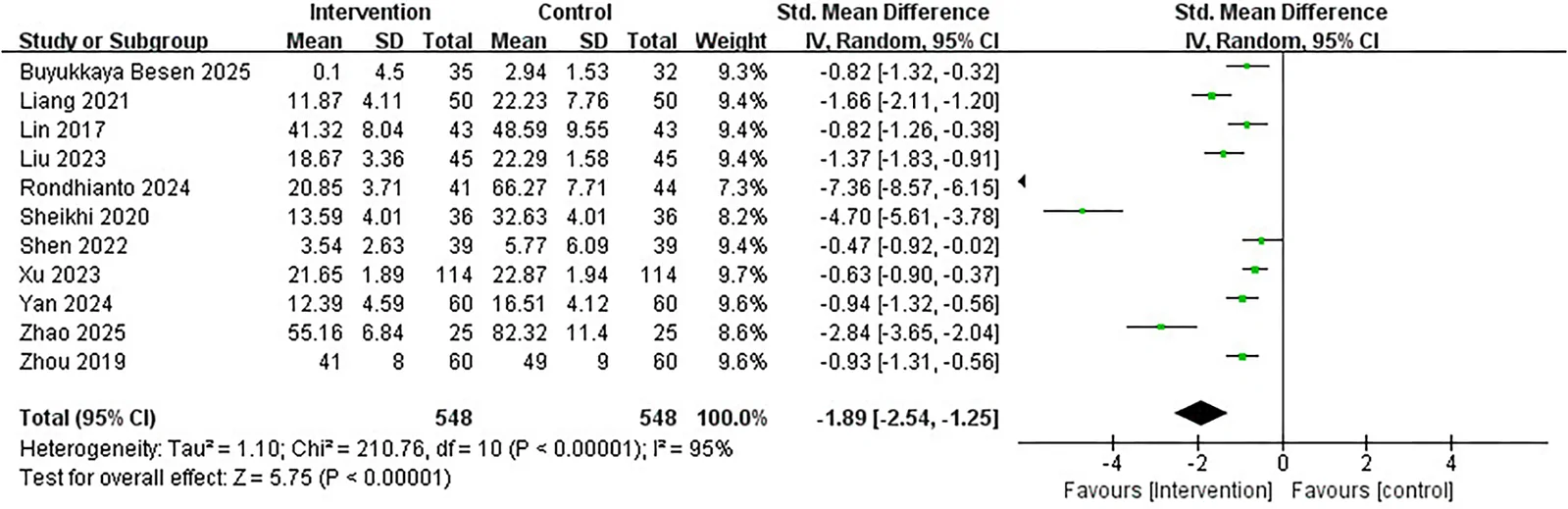
Forest plot of the overall effect on FoH scores. Standardised mean differences were pooled using a random-effects model. Negative values favour educational and behavioural interventions over control conditions.

Substantial heterogeneity was observed (Tau² = 1.10; Chi² = 210.76, df = 10, *p* < 0.00001; I² = 95%). The 95% prediction interval was wide and crossed the line of no effect (95% PI −4.34 to 0.56). This suggests that, although the pooled average effect favoured the intervention group, the true effect in a future similar trial may be smaller or non-significant. Given the substantial heterogeneity, this pooled SMD was interpreted as an exploratory average effect rather than as an estimate of a common intervention effect.

#### 3.5.2 Subgroup analysis by follow-up duration.

Follow-up duration was examined as a potential source of heterogeneity. Studies were grouped into short-term follow-up (≤3 months) and longer follow-up (>3 months). This analysis was exploratory because one trial [35] contributed separate short-term and longer-term estimates; therefore, the subgroup findings were interpreted cautiously.

In the short-term follow-up group, the pooled effect favoured the intervention group (SMD = −2.16; 95% CI −3.01 to −1.32; *p* < 0.00001), with substantial heterogeneity (I² = 96%). In the longer follow-up group, the pooled effect was in the same direction (SMD = −2.25; 95% CI −3.84 to −0.65; *p* = 0.006), also with substantial heterogeneity (I² = 97%). The test for subgroup differences was not statistically significant (χ² = 0.01, df = 1, *p* = 0.93), providing no statistical evidence that effects differed by follow-up duration. These findings are summarised in Fig 4.

**Fig 4:**
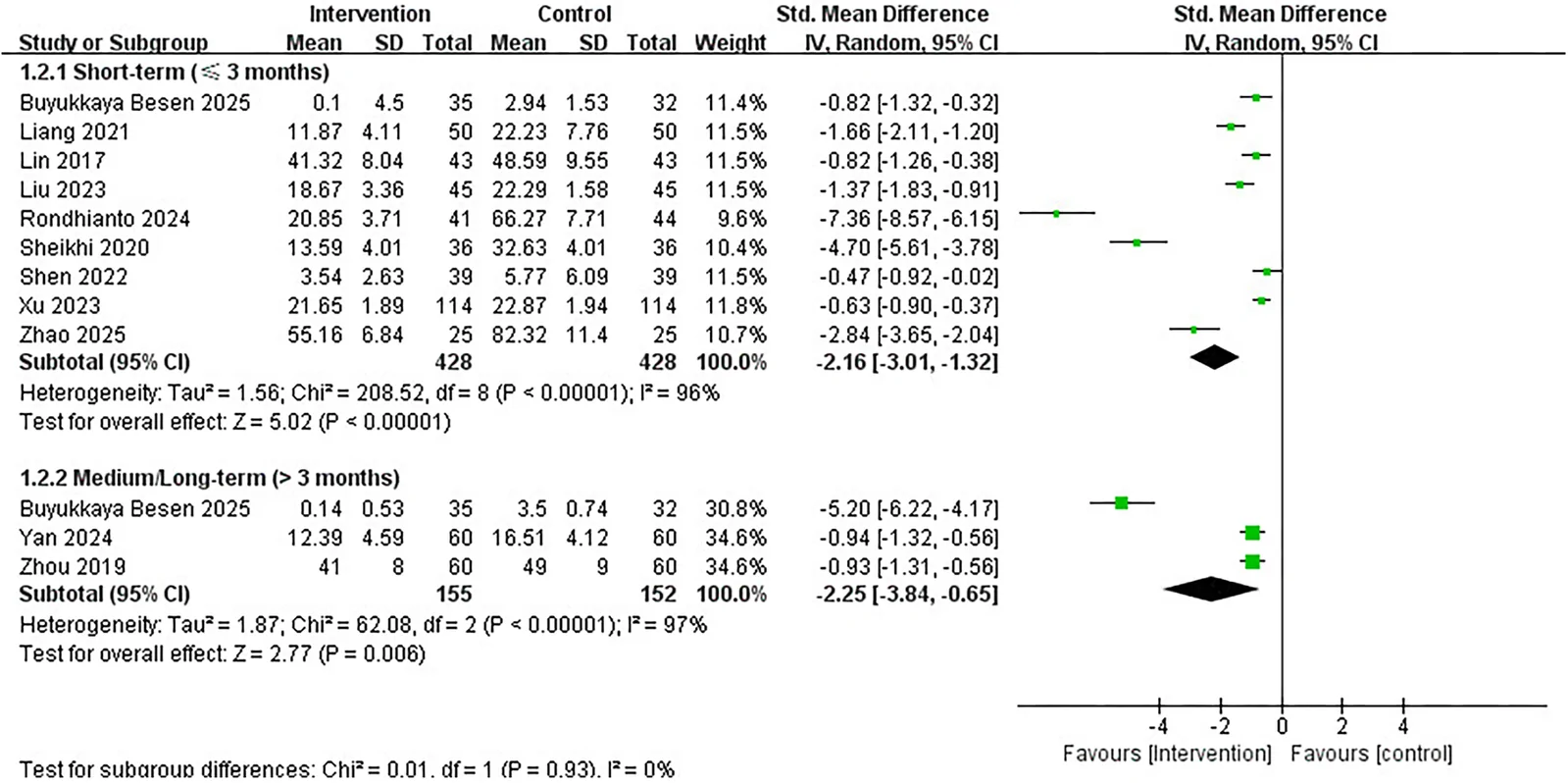
Subgroup analysis of FoH outcomes by follow-up duration. Studies were grouped as short-term follow-up (≤3 months) or longer follow-up (>3 months). The subgroup analysis was exploratory.

#### 3.5.3 Subgroup analysis by intervention type.

Intervention category was also examined as a potential source of heterogeneity. Nursing theory or education-based interventions showed a pooled effect favouring the intervention group (SMD = −1.61; 95% CI −2.32 to −0.90; *p* < 0.00001), with substantial heterogeneity (I² = 95%). Psychological or behavioural theory-based interventions showed a similar direction of effect (SMD = −2.37; 95% CI −3.90 to −0.84; *p* = 0.002), with substantial heterogeneity (I² = 96%).

The test for subgroup differences was not statistically significant (χ² = 0.79, df = 1, *p* = 0.38), indicating no statistical evidence of differential effects between the two intervention categories. Because substantial heterogeneity persisted within both subgroups, intervention category did not fully explain between-study variability. These findings are summarised in Fig 5.

**Fig 5:**
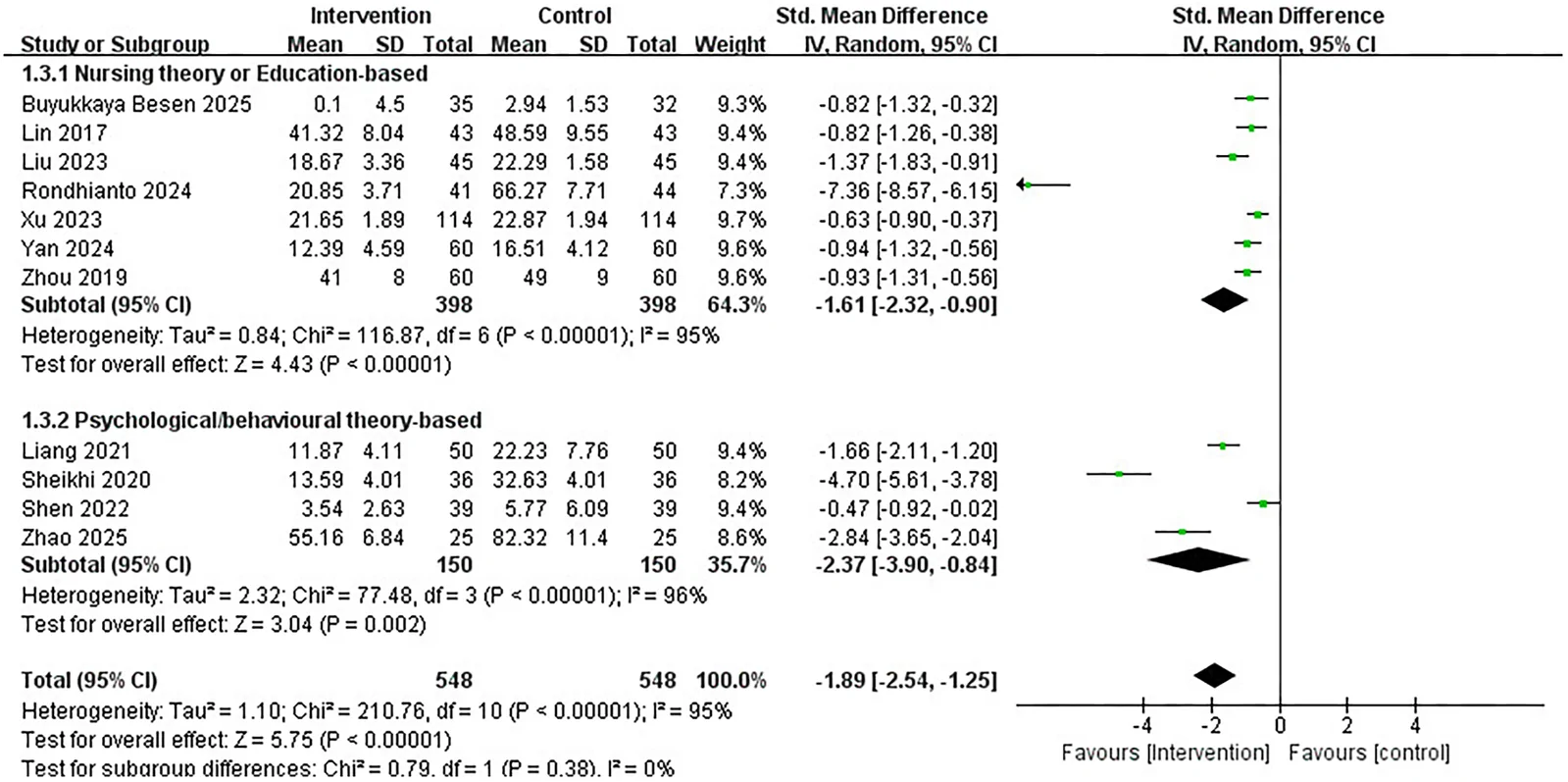
Subgroup analysis of FoH outcomes by intervention type. Interventions were grouped as nursing or education-based interventions and psychological or behavioural theory-based interventions.

#### 3.5.4 Sensitivity and additional robustness analyses.

A leave-one-out sensitivity analysis was conducted to assess whether the pooled effect was influenced by any single study. Sequentially excluding each study showed that the pooled effect remained statistically significant and in the same direction in all iterations.

The largest influence was observed when Rondhianto et al. [33] was excluded. The pooled effect was attenuated but remained statistically significant (SMD = −1.42; 95% CI −1.90 to −0.93), and heterogeneity decreased from 95% to 91.6% (Tau² = 0.54). Excluding Sheikhi et al. [34] also attenuated the pooled effect, but the result remained statistically significant (SMD = −1.60; 95% CI −2.18 to −1.02; I² = 94.0%; Tau² = 0.79). These findings suggest that the direction of the pooled effect was not dependent on a single study, although large-effect studies contributed to the magnitude of the pooled estimate and to between-study heterogeneity.

A post hoc sensitivity analysis was conducted by excluding studies published in Chinese-language domestic journals with limited international visibility. After excluding these studies, the pooled effect remained in favour of the intervention group (k = 6; n = 472; SMD = −2.77; 95% CI −4.23 to −1.31), but heterogeneity remained substantial (I² = 97.2%). When Chinese-language domestic journal studies were analysed separately, the pooled effect was smaller than the overall estimate but remained in favour of the intervention group (k = 5; n = 624; SMD = −1.06; 95% CI −1.43 to −0.69; I² = 78.3%).

Exploratory geographic sensitivity analyses were also conducted. Among China-based studies, the pooled effect favoured the intervention group (k = 8; n = 872; SMD = −1.13; 95% CI −1.50 to −0.76; I² = 84.1%). Among non-China studies, the pooled effect was also in favour of the intervention group but was based on only three trials and showed very substantial heterogeneity (k = 3; n = 224; SMD = −4.26; 95% CI −8.14 to −0.39; I² = 98.4%). These post hoc analyses did not suggest that the overall pooled effect was primarily driven by Chinese-language domestic journal studies, but they reinforced the need for caution because heterogeneity and geographic concentration persisted.

#### 3.5.5 Small-study effects and publication bias.

The funnel plot for the main analysis showed visual asymmetry, with smaller studies tending to show larger effect estimates (S1 Fig). Egger’s regression test also suggested significant asymmetry (p < 0.001), indicating possible small-study effects or publication bias.

Trim-and-fill analyses were performed as exploratory assessments of the potential influence of small-study effects. The primary L0 estimator imputed no missing studies and did not change the pooled estimate. However, an exploratory R0 estimator imputed two hypothetical missing studies and attenuated the pooled estimate (adjusted SMD = −1.14; 95% CI −1.90 to −0.37). These estimator-dependent findings suggest that small-study effects may have inflated the magnitude of the pooled effect, although the direction of effect remained in favour of the intervention in the exploratory adjusted analysis. These findings were considered in the GRADE assessment.

### 3.6 Certainty of evidence

The certainty of evidence for the primary outcome was assessed using the GRADE approach and is summarised in Table 2, with detailed downgrade decisions provided in S4 File. The certainty of evidence was rated as very low. Evidence was downgraded by one level for risk of bias because all included trials raised some concerns in the RoB 2 assessment, mainly due to self-reported FoH outcomes in trials where blinding of participants was generally not feasible.

**Table 2:** GRADE summary of findings for the effect of educational and behavioural interventions on FoH.

Outcome	No. of studies	No. of participants	Study design	Effect estimate	Risk of bias	Inconsistency	Indirectness	Imprecision	Publication bias	Certainty of evidence
Fear of hypoglycaemia score	11	1,096	Randomised controlled trials	SMD = −1.89; 95% CI −2.54 to −1.25; 95% PI −4.34 to 0.56	Serious^a^	Serious^b^	Serious^c^	Not serious^d^	Serious^e^	⊕○○○ Very low

*Note:* CI, confidence interval; FoH, fear of hypoglycaemia; GRADE, Grading of Recommendations Assessment, Development and Evaluation; PI, prediction interval; SMD, standardised mean difference.

^a^Downgraded one level for risk of bias because all included trials were judged as raising some concerns overall in the RoB 2 assessment. FoH was assessed using self-reported instruments, and blinding of participants was generally not feasible in educational, behavioural, psychological, nursing-led, family-based, or digitally supported interventions.

^b^Downgraded one level for inconsistency because substantial heterogeneity was observed across trials (Tau² = 1.10; I² = 95%; χ² = 210.76, df = 10, p < 0.00001). The 95% prediction interval crossed the line of no effect, indicating that the true effect in future similar studies may vary substantially.

^c^Downgraded one level for indirectness because most included studies were conducted in China, several were published in Chinese-language domestic journals with limited international visibility, and interventions differed substantially in theoretical basis, delivery format, intensity, and healthcare context. These factors limited the generalisability of the pooled estimate to broader populations and healthcare systems.

^d^Not downgraded for imprecision because the pooled 95% CI did not cross the line of no effect. The uncertainty reflected by the wide prediction interval was considered under inconsistency.

^e^Downgraded one level for suspected publication bias because funnel plot asymmetry and Egger’s regression test suggested possible small-study effects or publication bias (p < 0.001). Trim-and-fill findings were estimator-dependent and suggested possible attenuation of the pooled estimate in exploratory analysis.

Evidence was also downgraded by one level for inconsistency because substantial heterogeneity was observed across trials (Tau² = 1.10; I² = 95%), and the 95% prediction interval crossed the line of no effect. Evidence was further downgraded by one level for indirectness because the evidence base was geographically concentrated, most studies were conducted in China, several studies were published in Chinese-language domestic journals with limited international visibility, and interventions differed substantially in theoretical basis, delivery format, intensity, and healthcare context. These factors limited the generalisability of the pooled estimate to broader populations and healthcare systems.

No downgrade was applied for imprecision because the pooled 95% CI did not cross the line of no effect. The uncertainty reflected by the wide prediction interval was considered under inconsistency. A further downgrade was applied for suspected publication bias because funnel plot asymmetry, Egger’s regression test, and estimator-dependent trim-and-fill findings suggested possible small-study effects. Therefore, although the exploratory pooled average effect favoured the intervention group, confidence in the magnitude, consistency, and generalisability of the effect was very limited.

## 4. Discussion

### 4.1 Main findings

This review found very low-certainty evidence that structured educational and behavioural interventions may reduce fear of hypoglycaemia (FoH) in adults with type 2 diabetes mellitus (T2DM). Across 11 randomised controlled trials involving 1,096 participants from four countries, the exploratory pooled average effect favoured the intervention group (SMD = −1.89; 95% CI −2.54 to −1.25). However, this estimate should be interpreted cautiously because heterogeneity was substantial (Tau² = 1.10; I² = 95%), the 95% prediction interval crossed the line of no effect (95% PI −4.34 to 0.56), funnel plot asymmetry and Egger’s test suggested possible small-study effects, and the trim-and-fill findings were estimator-dependent. Overall, the findings suggest possible benefit, but confidence in the magnitude, consistency, and generalisability of the pooled effect is very limited.

### 4.2 Population and cultural context

Most included studies were conducted in China, with the remaining trials from Indonesia, Iran, and Turkey. This geographic concentration may limit the applicability of the findings to broader healthcare settings, including Western and more culturally diverse populations. It also points to the role of local care models in shaping intervention delivery. Several Chinese studies used structured nursing or education-based approaches, which may reflect clinical practice patterns in high-volume diabetes care settings. Participant characteristics differed across trials, including individuals with elevated baseline FoH [27,28,32,34], those newly initiated on insulin therapy [35], and those with impaired awareness of hypoglycaemia [26]. These differences may have contributed to variation in intervention effects.

The additional sensitivity analyses provided further context for interpreting the geographic concentration of the evidence. Excluding studies published in Chinese-language domestic journals with limited international visibility did not reverse the direction of effect, but heterogeneity remained very high. When these studies were analysed separately, the pooled effect was smaller than the overall estimate. This suggests that the overall pooled effect was not primarily driven by Chinese-language domestic journal studies alone. However, the evidence base remained geographically concentrated, and the exploratory comparison between China-based and non-China studies was limited by the small number of non-China trials and very substantial heterogeneity. These considerations were reflected in the GRADE assessment through downgrading for indirectness.

### 4.3 Clinical interpretation and implications

The pooled estimate suggests that FoH may respond to structured educational and behavioural support in adults with T2DM. This interpretation is clinically relevant because FoH has been linked to poorer diabetes self-management and compensatory behaviours that may interfere with glycaemic control [36,37]. The size of the pooled effect should not be read as a uniform or definitive intervention effect, as the prediction interval crossed the line of no effect and indicated that future trials in similar populations may show smaller or non-significant effects.

Studies in type 1 diabetes have reported variable and often modest effects of educational, psychological, or technology-supported interventions on FoH [16,17,38,39]. Direct comparison with the present review is not appropriate because diabetes type, baseline FoH severity, treatment regimens, technology use, and intervention content differ across populations. In adults with T2DM, FoH may be closely linked to self-management confidence, previous hypoglycaemia experience, and access to diabetes education and support [10]. This explanation remains tentative and requires direct testing in trials designed to measure these pathways.

The relatively large pooled SMD observed in the present review should not be interpreted as evidence that educational and behavioural interventions are more effective for FoH in T2DM than in T1DM. Several factors may have contributed to the larger estimate, including the small number of included trials, substantial heterogeneity, self-reported outcomes in trials where participant blinding was generally not feasible, possible small-study effects, differences in FoH measurement tools, and contextual concentration of the evidence base. Therefore, the clinical implication of this review is not that a large and consistent effect has been established, but that structured educational and behavioural approaches may be promising and warrant more rigorous evaluation in adults with T2DM.

Educational and behavioural interventions may be relevant in settings where access to specialist psychological services or advanced diabetes technologies is limited. In the included studies, these interventions were commonly delivered through nurse-led, family-based, or digitally supported formats. However, implementation costs, cost-effectiveness, and health-system readiness were not assessed in this review. The feasibility and scalability of these approaches therefore require direct evaluation before conclusions can be drawn about their use in resource-limited settings [40,41].

### 4.4 Theoretical frameworks and possible mechanisms

The subgroup analysis by intervention category did not provide statistical evidence that effects differed between nursing or education-based interventions and psychological or behavioural theory-based interventions (*p* = 0.38). Although the point estimate was larger for psychological and behavioural theory-based interventions, heterogeneity remained substantial within both subgroups. The current evidence therefore does not support a clear conclusion about the superiority of one intervention category over another.

The intervention category in this review was intentionally broad because the review focused on structured educational and behavioural interventions addressing FoH, hypoglycaemia-related worry, or avoidance in adults with T2DM. However, this broad category included heterogeneous approaches, such as psychological therapy, traditional Chinese medicine-based emotional nursing, family caregiver empowerment, nursing-led education, theory-informed health education, and digital clinic support. Although these interventions shared a broad aim of reducing FoH through education, behavioural support, emotional regulation, or self-management reinforcement, they should not be interpreted as a single uniform intervention model. This heterogeneity supports the use of cautious narrative interpretation alongside the exploratory quantitative synthesis.

Interventions grouped under different theoretical categories may have shared several components. Psychological approaches, such as stress inoculation training [28] and eye movement desensitisation and reprocessing [34], appeared to address cognitive and emotional responses to hypoglycaemia more directly. Interventions informed by Hope Theory [29], King’s Theory [25], or the Common-Sense Model [26] were more often delivered through structured education, motivational support, or behavioural guidance. Across categories, several programmes included psychoeducation, emotional support, self-management reinforcement, family involvement, or follow-up contact. These shared elements may partly explain why subgroup differences were not clearly observed, although the small number of studies limits interpretation.

The available evidence is insufficient to identify which components were responsible for reductions in FoH. Few studies described in detail how theoretical models were translated into intervention content, and only a minority assessed potential pathways such as empowerment, coping style, or self-management confidence [26,28,35]. Any explanation of mechanisms should therefore be considered hypothesis-generating. Future trials would be strengthened by clearer reporting of intervention components and fidelity, together with process evaluations or mediation analyses where feasible.

### 4.5 Effects across follow-up durations

The subgroup analysis by follow-up duration found statistically significant pooled effects in both short-term follow-up and follow-up beyond 3 months. However, the difference between follow-up subgroups was not statistically significant (p = 0.93), and substantial heterogeneity persisted in both subgroups. One trial also contributed separate short-term and longer-term estimates to this exploratory subgroup analysis. For these reasons, the subgroup analysis should be regarded as descriptive and hypothesis-generating rather than as evidence that intervention effects were sustained over time.

Evidence on durability remains limited. Only a small number of studies assessed outcomes up to approximately 6 months [25,30,35], and no included trial reported follow-up beyond one year. Longer follow-up is needed to determine whether reductions in FoH are maintained and whether booster sessions, continued contact, or integration into routine diabetes care are required.

## 5. Strengths and limitations

This review has several strengths. It focused specifically on adults with T2DM and included only randomised controlled trials. The review was prospectively registered, followed PRISMA 2020 guidance, and used RoB 2 and GRADE to assess risk of bias and certainty of evidence. The analysis used a random-effects model, reported Tau² and a prediction interval, and conducted subgroup and leave-one-out sensitivity analyses. Additional trim-and-fill, journal and geographic sensitivity analyses, were conducted to examine small-study effects, contextual concentration, and robustness of the findings. A TIDieR-style supplementary table is provided to improve transparency of intervention reporting. Study-level extracted data and additional analyses are provided in the Supporting Information files and are available in Zenodo.

Several limitations should be considered. First, heterogeneity was substantial (Tau² = 1.10; I² = 95%), and the prediction interval crossed the line of no effect. The pooled estimate therefore should not be interpreted as a consistent effect across all settings. Differences in intervention content, baseline FoH severity, participant characteristics, follow-up duration, delivery format, measurement tools, and cultural context may have contributed to this variability.

Second, the certainty of evidence was rated as very low. This rating was driven by risk of bias concerns, substantial inconsistency, indirectness, and suspected publication bias or small-study effects. Blinding of participants and intervention providers was generally not feasible because the interventions were educational or behavioural, and FoH was assessed using self-reported instruments. These features may have influenced outcome measurement.

Third, the included studies were geographically concentrated in Asian countries, especially China. Several trials were published in Chinese-language domestic journals with limited international visibility. Although sensitivity analyses did not suggest that these studies alone explained the overall pooled estimate, the concentration of evidence in specific healthcare and cultural contexts limits generalisability to other settings.

Fourth, the intervention category was broad and heterogeneous. Included interventions ranged from psychological therapy and stress inoculation training to nursing-led education, traditional Chinese medicine-based emotional nursing, family caregiver empowerment, and digital support. Although these approaches shared a broad educational, behavioural, and FoH-related focus, the available evidence is insufficient to identify which components were responsible for changes in FoH. Intervention fidelity, adherence, tailoring, and mechanisms of change were also incompletely reported in several studies.

Finally, funnel plot asymmetry, Egger’s regression test, and estimator-dependent trim-and-fill findings suggested possible small-study effects. Although asymmetry may also reflect true heterogeneity across complex interventions, it further supports cautious interpretation of the magnitude of the pooled effect.

## 6. Conclusion

Very low-certainty evidence suggests that structured educational and behavioural interventions may reduce FoH among adults with T2DM. However, the magnitude and generalisability of this effect remain uncertain because heterogeneity was substantial, the prediction interval crossed the line of no effect, and possible small-study effects were observed. Future RCTs should use standardised FoH measures, report intervention components and fidelity more clearly, include longer follow-up, and examine mechanisms of change through process evaluation or mediation analysis where feasible.

## Supporting information

S1 Fig Funnel plot for the main meta-analysis of FoH outcomes. The plot displays study-level standardised mean differences against their standard errors. Visual asymmetry was observed and further assessed using Egger’s regression test.
(TIF)

S1 Checklist PRISMA 2020 checklist. This checklist reports the manuscript sections corresponding to the PRISMA 2020 items.
(DOCX)

S1 File Full search strategies. This file provides the complete search strategies used for all databases.
(DOCX)

S2 File Extracted dataset and additional analyses used for meta-analysis. This file contains the study-level aggregate data used for the main random-effects meta-analysis, prediction interval, leave-one-out sensitivity analysis, subgroup analyses, and publication bias assessment.
(XLSX)

S3 File Risk of bias assessment using RoB 2. This file provides the domain-level risk of bias judgements and supporting reasons for all included RCTs.
(XLSX)

S4 File Detailed GRADE assessment. This file presents the certainty of evidence assessment for the primary outcome, including downgrade decisions and supporting explanations.
(XLSX)

S1 Table TIDieR-style summary of intervention characteristics. This table summarises the rationale, materials, providers, delivery mode, setting, intensity, duration, tailoring, comparator, and fidelity/adherence reporting of the included interventions.
(DOCX)
