# Supplementary material for: Educational and behavioural interventions for reducing fear of hypoglycaemia in adults with type 2 diabetes: A systematic review and meta-analysis of randomised controlled trials
Source: PLoS One. 2026 Oct 5;21(10):e0358881. doi: 10.1371/journal.pone.0358881 (PMC13637911; doi:10.1371/journal.pone.0358881)
Supplement: Supplementary file 1 [file pone.0358881.s002.docx]

**S1 Checklist. PRISMA 2020 checklist**

*Concise location version for manuscript submission*

This checklist identifies where each PRISMA 2020 item is reported in the manuscript or Supporting Information. It is intentionally concise and does not reproduce manuscript text.

| **Section/topic** | **Item** | **Checklist item** | **Location reported** |
| --- | --- | --- | --- |
| **TITLE** | 1 | Identify the report as a systematic review. | Title page |
| **ABSTRACT** | 2 | See the PRISMA 2020 for Abstracts checklist. | Abstract |
| **INTRODUCTION** | 3 | Describe the rationale for the review in the context of existing knowledge. | Introduction, paragraphs 1-3 |
| **INTRODUCTION** | 4 | Provide an explicit statement of the objective(s) or question(s) the review addresses. | Introduction, final paragraph |
| **METHODS** | 5 | Specify inclusion/exclusion criteria and how studies were grouped for syntheses. | Methods, section 2.2; section 2.6 |
| **METHODS** | 6 | Specify information sources searched and the date each source was last searched. | Methods, section 2.1 |
| **METHODS** | 7 | Present full search strategies, including filters and limits. | Methods, section 2.1; S1 File |
| **METHODS** | 8 | Specify the study selection process, including reviewers and independent screening. | Methods, section 2.3 |
| **METHODS** | 9 | Specify the data collection process, including reviewers and independent extraction. | Methods, section 2.4 |
| **METHODS** | 10a | List outcomes sought and how eligible results/time points were selected. | Methods, sections 2.2 and 2.4 |
| **METHODS** | 10b | List other variables sought and assumptions for missing or unclear information. | Methods, section 2.4; S2 File |
| **METHODS** | 11 | Specify risk of bias methods, tool, reviewers, and independent assessment. | Methods, section 2.5; S3 File |
| **METHODS** | 12 | Specify effect measures used in synthesis or presentation. | Methods, section 2.6 |
| **METHODS** | 13a | Describe how studies were selected for each synthesis. | Methods, sections 2.2, 2.4, and 2.6 |
| **METHODS** | 13b | Describe data preparation, missing summary statistics, and conversions. | Methods, section 2.4; S2 File |
| **METHODS** | 13c | Describe tabular or visual display of individual study and synthesis results. | Methods, section 2.6; Table 1; Figures 3-5 |
| **METHODS** | 13d | Describe synthesis methods, model, heterogeneity assessment, and software. | Methods, section 2.6 |
| **METHODS** | 13e | Describe methods to explore possible causes of heterogeneity. | Methods, section 2.6 |
| **METHODS** | 13f | Describe sensitivity analyses conducted. | Methods, section 2.6 |
| **METHODS** | 14 | Describe methods to assess reporting bias/missing results. | Methods, section 2.6 |
| **METHODS** | 15 | Describe methods to assess certainty/confidence in the evidence. | Methods, section 2.5; S4 File |
| **RESULTS** | 16a | Describe search and selection results, ideally with a flow diagram. | Results, section 3.1; Figure 1 |
| **RESULTS** | 16b | Cite apparently eligible studies excluded and explain why. | Results, section 3.1; Figure 1 |
| **RESULTS** | 17 | Cite each included study and present characteristics. | Results, section 3.2; Table 1 |
| **RESULTS** | 18 | Present risk of bias assessments for included studies. | Results, section 3.3; Figure 2; S3 File |
| **RESULTS** | 19 | Present study-level summary statistics/effect estimates with precision where appropriate. | Results, sections 3.4-3.5; Figure 3; S2 File |
| **RESULTS** | 20a | Summarise characteristics and risk of bias among studies in each synthesis. | Results, sections 3.2-3.5 |
| **RESULTS** | 20b | Present all statistical syntheses, including estimates, precision, heterogeneity, and direction. | Results, section 3.5; Figures 3-5 |
| **RESULTS** | 20c | Present investigations of possible causes of heterogeneity. | Results, sections 3.5.2 and 3.5.3 |
| **RESULTS** | 20d | Present all sensitivity analyses. | Results, section 3.5.4; S2 File |
| **RESULTS** | 21 | Present reporting bias assessments for each synthesis assessed. | Results, section 3.5.5; Supplementary Figure 1 |
| **RESULTS** | 22 | Present certainty/confidence assessments for each outcome. | Results, section 3.6; Table 2; S4 File |
| **DISCUSSION** | 23a | Provide a general interpretation in the context of other evidence. | Discussion, sections 4.1-4.3 |
| **DISCUSSION** | 23b | Discuss limitations of the evidence included. | Discussion, section 5 |
| **DISCUSSION** | 23c | Discuss limitations of the review process. | Discussion, section 5 |
| **DISCUSSION** | 23d | Discuss implications for practice, policy, and future research. | Discussion, sections 4.3-4.5; Conclusion |
| **OTHER INFORMATION** | 24a | Provide registration information, register name, and registration number. | Methods, section 2 |
| **OTHER INFORMATION** | 24b | Indicate where the protocol can be accessed or state that none was prepared. | Methods, section 2; PROSPERO CRD42025630957 |
| **OTHER INFORMATION** | 24c | Describe any protocol amendments. | Not applicable; no protocol amendments reported |
| **OTHER INFORMATION** | 25 | Describe financial/non-financial support and funder role. | Funding statement |
| **OTHER INFORMATION** | 26 | Declare competing interests. | Competing interests statement |
| **OTHER INFORMATION** | 27 | Report availability of data, code, and materials. | Data Availability statement; S2 File |

Source: Page MJ, McKenzie JE, Bossuyt PM, Boutron I, Hoffmann TC, Mulrow CD, et al. The PRISMA 2020 statement: an updated guideline for reporting systematic reviews. BMJ. 2021;372:n71. doi:10.1136/bmj.n71. Licensed under CC BY 4.0.
