# Supplementary material for: Educational and behavioural interventions for reducing fear of hypoglycaemia in adults with type 2 diabetes: A systematic review and meta-analysis of randomised controlled trials
Source: PLoS One. 2026 Oct 5;21(10):e0358881. doi: 10.1371/journal.pone.0358881 (PMC13637911; doi:10.1371/journal.pone.0358881)
Supplement: Supplementary file 2 [file pone.0358881.s003.docx]

**S2 File. Full search strategies**

This file provides the full search strategies used for the systematic review and meta-analysis of educational and behavioural interventions for reducing fear of hypoglycaemia among adults with type 2 diabetes mellitus. Searches were conducted from database inception to 31 December 2025. A bridge search was conducted on 20 April 2026 to identify newly published eligible trials.

The search strategy combined terms related to hypoglycaemia, fear or psychological distress, type 2 diabetes mellitus, educational or behavioural interventions, and randomised controlled trials. Search terms were adapted for each database according to database-specific indexing terms and search syntax. Only randomised controlled trials were eligible for inclusion. No additional eligible trial meeting the inclusion criteria was identified in the bridge search.

| **Database** | **Search period** | **Search strategy** | **Limits / notes** |
| --- | --- | --- | --- |
| **PubMed** | Inception to December 2025; bridge search on 20 April 2026 | ("Hypoglycemia"[Mesh] OR hypoglyc*[Title/Abstract] OR hypoglycaemia[Title/Abstract] OR hypoglycemia[Title/Abstract] OR "low blood sugar"[Title/Abstract] OR "low glucose"[Title/Abstract] OR glycopenia[Title/Abstract] OR glucopenia[Title/Abstract] OR "low serum glucose"[Title/Abstract] OR "blood glucose reduction"[Title/Abstract] OR "reactive hypoglycemia"[Title/Abstract] OR "reactive hypoglycaemia"[Title/Abstract] OR "fasting hypoglycemia"[Title/Abstract] OR "fasting hypoglycaemia"[Title/Abstract] OR "postprandial hypoglycemia"[Title/Abstract] OR "postprandial hypoglycaemia"[Title/Abstract]) AND ("Fear"[Mesh] OR "Anxiety"[Mesh] OR "Psychological Distress"[Mesh] OR fear*[Title/Abstract] OR anxiet*[Title/Abstract] OR concern*[Title/Abstract] OR worry*[Title/Abstract] OR stress[Title/Abstract] OR distress[Title/Abstract] OR burden*[Title/Abstract] OR "psychological burden"[Title/Abstract] OR "emotional distress"[Title/Abstract] OR "fear of hypoglycemia"[Title/Abstract] OR "fear of hypoglycaemia"[Title/Abstract] OR "hypoglycemia fear"[Title/Abstract] OR "hypoglycaemia fear"[Title/Abstract]) AND ("Diabetes Mellitus, Type 2"[Mesh] OR "type 2 diabetes"[Title/Abstract] OR "type II diabetes"[Title/Abstract] OR T2DM[Title/Abstract] OR NIDDM[Title/Abstract] OR "adult-onset diabetes"[Title/Abstract] OR "non-insulin dependent diabetes"[Title/Abstract] OR "non-insulin-dependent diabetes"[Title/Abstract] OR "maturity-onset diabetes"[Title/Abstract]) AND ("Therapy"[Mesh] OR "Health Education"[Mesh] OR intervention*[Title/Abstract] OR treatment*[Title/Abstract] OR management[Title/Abstract] OR program*[Title/Abstract] OR programme*[Title/Abstract] OR education*[Title/Abstract] OR training[Title/Abstract] OR counselling[Title/Abstract] OR counseling[Title/Abstract] OR "psychological intervention*"[Title/Abstract] OR "behavioural intervention*"[Title/Abstract] OR "behavioral intervention*"[Title/Abstract] OR "educational intervention*"[Title/Abstract] OR "educational program*"[Title/Abstract] OR "educational programme*"[Title/Abstract] OR "psychosocial therapy"[Title/Abstract] OR "lifestyle modification"[Title/Abstract] OR "self-management"[Title/Abstract] OR "nursing intervention*"[Title/Abstract]) AND ("Randomized Controlled Trial"[Publication Type] OR "Controlled Clinical Trial"[Publication Type] OR "Randomized Controlled Trials as Topic"[Mesh] OR randomized[Title/Abstract] OR randomised[Title/Abstract] OR randomly[Title/Abstract] OR placebo[Title/Abstract] OR trial[Title/Abstract]) | RCT-related terms included because only randomised controlled trials were eligible. |
| **Cochrane Library** | Inception to December 2025; bridge search on 20 April 2026 | (MeSH descriptor: [Hypoglycemia] explode all trees OR hypoglyc* OR hypoglycaemia OR hypoglycemia OR "low blood sugar" OR "low glucose" OR glycopenia OR glucopenia OR "low serum glucose" OR "blood glucose reduction" OR "reactive hypoglycemia" OR "reactive hypoglycaemia" OR "fasting hypoglycemia" OR "fasting hypoglycaemia" OR "postprandial hypoglycemia" OR "postprandial hypoglycaemia":ti,ab,kw) AND (MeSH descriptor: [Fear] explode all trees OR MeSH descriptor: [Anxiety] explode all trees OR MeSH descriptor: [Psychological Distress] explode all trees OR fear* OR anxiet* OR concern* OR worry* OR stress OR distress OR burden* OR "psychological burden" OR "emotional distress" OR "fear of hypoglycemia" OR "fear of hypoglycaemia" OR "hypoglycemia fear" OR "hypoglycaemia fear":ti,ab,kw) AND (MeSH descriptor: [Diabetes Mellitus, Type 2] explode all trees OR "type 2 diabetes" OR "type II diabetes" OR T2DM OR NIDDM OR "adult-onset diabetes" OR "non-insulin dependent diabetes" OR "non-insulin-dependent diabetes" OR "maturity-onset diabetes":ti,ab,kw) AND (MeSH descriptor: [Therapy] explode all trees OR MeSH descriptor: [Health Education] explode all trees OR intervention* OR treatment* OR management OR program* OR programme* OR education* OR training OR counselling OR counseling OR "psychological intervention*" OR "behavioural intervention*" OR "behavioral intervention*" OR "educational intervention*" OR "educational program*" OR "educational programme*" OR "psychosocial therapy" OR "lifestyle modification" OR "self-management" OR "nursing intervention*":ti,ab,kw) AND (randomized OR randomised OR randomly OR placebo OR trial:ti,ab,kw) | Search terms adapted for Cochrane Library syntax. |
| **Embase** | Inception to December 2025; bridge search on 20 April 2026 | ('hypoglycemia'/exp OR hypoglyc*:ti,ab,kw OR hypoglycaemia:ti,ab,kw OR hypoglycemia:ti,ab,kw OR 'low blood sugar':ti,ab,kw OR 'low glucose':ti,ab,kw OR glycopenia:ti,ab,kw OR glucopenia:ti,ab,kw OR 'low serum glucose':ti,ab,kw OR 'blood glucose reduction':ti,ab,kw OR 'reactive hypoglycemia':ti,ab,kw OR 'reactive hypoglycaemia':ti,ab,kw OR 'fasting hypoglycemia':ti,ab,kw OR 'fasting hypoglycaemia':ti,ab,kw OR 'postprandial hypoglycemia':ti,ab,kw OR 'postprandial hypoglycaemia':ti,ab,kw) AND ('fear'/exp OR 'anxiety'/exp OR 'psychological distress'/exp OR fear*:ti,ab,kw OR anxiet*:ti,ab,kw OR concern*:ti,ab,kw OR worry*:ti,ab,kw OR stress:ti,ab,kw OR distress:ti,ab,kw OR burden*:ti,ab,kw OR 'psychological burden':ti,ab,kw OR 'emotional distress':ti,ab,kw OR 'fear of hypoglycemia':ti,ab,kw OR 'fear of hypoglycaemia':ti,ab,kw OR 'hypoglycemia fear':ti,ab,kw OR 'hypoglycaemia fear':ti,ab,kw) AND ('type 2 diabetes mellitus'/exp OR 'type 2 diabetes':ti,ab,kw OR 'type II diabetes':ti,ab,kw OR T2DM:ti,ab,kw OR NIDDM:ti,ab,kw OR 'adult-onset diabetes':ti,ab,kw OR 'non-insulin dependent diabetes':ti,ab,kw OR 'non-insulin-dependent diabetes':ti,ab,kw OR 'maturity-onset diabetes':ti,ab,kw) AND ('therapy'/exp OR 'health education'/exp OR intervention*:ti,ab,kw OR treatment*:ti,ab,kw OR management:ti,ab,kw OR program*:ti,ab,kw OR programme*:ti,ab,kw OR education*:ti,ab,kw OR training:ti,ab,kw OR counselling:ti,ab,kw OR counseling:ti,ab,kw OR 'psychological intervention*':ti,ab,kw OR 'behavioural intervention*':ti,ab,kw OR 'behavioral intervention*':ti,ab,kw OR 'educational intervention*':ti,ab,kw OR 'educational program*':ti,ab,kw OR 'educational programme*':ti,ab,kw OR 'psychosocial therapy':ti,ab,kw OR 'lifestyle modification':ti,ab,kw OR 'self-management':ti,ab,kw OR 'nursing intervention*':ti,ab,kw) AND ('randomized controlled trial'/exp OR 'controlled clinical trial'/exp OR randomized:ti,ab,kw OR randomised:ti,ab,kw OR randomly:ti,ab,kw OR placebo:ti,ab,kw OR trial:ti,ab,kw) | Search terms adapted for Embase indexing and syntax. |
| **PsycINFO** | Inception to December 2025; bridge search on 20 April 2026 | (DE "Hypoglycemia" OR hypoglyc* OR hypoglycaemia OR hypoglycemia OR "low blood sugar" OR "low glucose" OR glycopenia OR glucopenia OR "low serum glucose" OR "blood glucose reduction" OR "reactive hypoglycemia" OR "reactive hypoglycaemia" OR "fasting hypoglycemia" OR "fasting hypoglycaemia" OR "postprandial hypoglycemia" OR "postprandial hypoglycaemia") AND (DE "Fear" OR DE "Anxiety" OR DE "Psychological Stress" OR fear* OR anxiet* OR concern* OR worry* OR stress OR distress OR burden* OR "psychological burden" OR "emotional distress" OR "fear of hypoglycemia" OR "fear of hypoglycaemia" OR "hypoglycemia fear" OR "hypoglycaemia fear") AND (DE "Diabetes Mellitus" OR "type 2 diabetes" OR "type II diabetes" OR T2DM OR NIDDM OR "adult-onset diabetes" OR "non-insulin dependent diabetes" OR "non-insulin-dependent diabetes" OR "maturity-onset diabetes") AND (DE "Treatment" OR DE "Health Education" OR intervention* OR treatment* OR management OR program* OR programme* OR education* OR training OR counselling OR counseling OR "psychological intervention*" OR "behavioural intervention*" OR "behavioral intervention*" OR "educational intervention*" OR "educational program*" OR "educational programme*" OR "psychosocial therapy" OR "lifestyle modification" OR "self-management" OR "nursing intervention*") AND (randomized OR randomised OR randomly OR placebo OR trial OR "randomized controlled trial" OR "randomised controlled trial" OR "controlled clinical trial") | Search terms adapted for PsycINFO syntax. |
| **Scopus** | Inception to December 2025; bridge search on 20 April 2026 | TITLE-ABS-KEY(hypoglyc* OR hypoglycaemia OR hypoglycemia OR "low blood sugar" OR "low glucose" OR glycopenia OR glucopenia OR "low serum glucose" OR "blood glucose reduction" OR "reactive hypoglycemia" OR "reactive hypoglycaemia" OR "fasting hypoglycemia" OR "fasting hypoglycaemia" OR "postprandial hypoglycemia" OR "postprandial hypoglycaemia") AND TITLE-ABS-KEY(fear* OR anxiet* OR concern* OR worry* OR stress OR distress OR burden* OR "psychological burden" OR "emotional distress" OR "fear of hypoglycemia" OR "fear of hypoglycaemia" OR "hypoglycemia fear" OR "hypoglycaemia fear") AND TITLE-ABS-KEY("type 2 diabetes" OR "type II diabetes" OR T2DM OR NIDDM OR "adult-onset diabetes" OR "non-insulin dependent diabetes" OR "non-insulin-dependent diabetes" OR "maturity-onset diabetes") AND TITLE-ABS-KEY(intervention* OR treatment* OR management OR program* OR programme* OR education* OR training OR counselling OR counseling OR "psychological intervention*" OR "behavioural intervention*" OR "behavioral intervention*" OR "educational intervention*" OR "educational program*" OR "educational programme*" OR "psychosocial therapy" OR "lifestyle modification" OR "self-management" OR "nursing intervention*") AND TITLE-ABS-KEY(randomized OR randomised OR randomly OR placebo OR trial OR "randomized controlled trial" OR "randomised controlled trial" OR "controlled clinical trial") | Search conducted in title, abstract, and keywords. |
| **Web of Science** | Inception to December 2025; bridge search on 20 April 2026 | TS=(hypoglyc* OR hypoglycaemia OR hypoglycemia OR "low blood sugar" OR "low glucose" OR glycopenia OR glucopenia OR "low serum glucose" OR "blood glucose reduction" OR "reactive hypoglycemia" OR "reactive hypoglycaemia" OR "fasting hypoglycemia" OR "fasting hypoglycaemia" OR "postprandial hypoglycemia" OR "postprandial hypoglycaemia") AND TS=(fear* OR anxiet* OR concern* OR worry* OR stress OR distress OR burden* OR "psychological burden" OR "emotional distress" OR "fear of hypoglycemia" OR "fear of hypoglycaemia" OR "hypoglycemia fear" OR "hypoglycaemia fear") AND TS=("type 2 diabetes" OR "type II diabetes" OR T2DM OR NIDDM OR "adult-onset diabetes" OR "non-insulin dependent diabetes" OR "non-insulin-dependent diabetes" OR "maturity-onset diabetes") AND TS=(intervention* OR treatment* OR management OR program* OR programme* OR education* OR training OR counselling OR counseling OR "psychological intervention*" OR "behavioural intervention*" OR "behavioral intervention*" OR "educational intervention*" OR "educational program*" OR "educational programme*" OR "psychosocial therapy" OR "lifestyle modification" OR "self-management" OR "nursing intervention*") AND TS=(randomized OR randomised OR randomly OR placebo OR trial OR "randomized controlled trial" OR "randomised controlled trial" OR "controlled clinical trial") | Search conducted using topic field. |
| **CINAHL** | Inception to December 2025; bridge search on 20 April 2026 | (MH "Hypoglycemia+" OR TI hypoglyc* OR AB hypoglyc* OR TI hypoglycaemia OR AB hypoglycaemia OR TI hypoglycemia OR AB hypoglycemia OR TI "low blood sugar" OR AB "low blood sugar" OR TI "low glucose" OR AB "low glucose" OR TI glycopenia OR AB glycopenia OR TI glucopenia OR AB glucopenia) AND (MH "Fear+" OR MH "Anxiety+" OR MH "Stress, Psychological+" OR TI fear* OR AB fear* OR TI anxiet* OR AB anxiet* OR TI concern* OR AB concern* OR TI worry* OR AB worry* OR TI stress OR AB stress OR TI distress OR AB distress OR TI burden* OR AB burden* OR TI "psychological burden" OR AB "psychological burden" OR TI "emotional distress" OR AB "emotional distress" OR TI "fear of hypoglycemia" OR AB "fear of hypoglycemia" OR TI "fear of hypoglycaemia" OR AB "fear of hypoglycaemia") AND (MH "Diabetes Mellitus, Type 2+" OR TI "type 2 diabetes" OR AB "type 2 diabetes" OR TI "type II diabetes" OR AB "type II diabetes" OR TI T2DM OR AB T2DM OR TI NIDDM OR AB NIDDM OR TI "adult-onset diabetes" OR AB "adult-onset diabetes" OR TI "non-insulin dependent diabetes" OR AB "non-insulin dependent diabetes" OR TI "non-insulin-dependent diabetes" OR AB "non-insulin-dependent diabetes") AND (MH "Therapeutics+" OR MH "Health Education+" OR TI intervention* OR AB intervention* OR TI treatment* OR AB treatment* OR TI management OR AB management OR TI program* OR AB program* OR TI programme* OR AB programme* OR TI education* OR AB education* OR TI training OR AB training OR TI counselling OR AB counselling OR TI counseling OR AB counseling OR TI "psychological intervention*" OR AB "psychological intervention*" OR TI "behavioural intervention*" OR AB "behavioural intervention*" OR TI "behavioral intervention*" OR AB "behavioral intervention*" OR TI "educational intervention*" OR AB "educational intervention*" OR TI "educational program*" OR AB "educational program*" OR TI "educational programme*" OR AB "educational programme*" OR TI "psychosocial therapy" OR AB "psychosocial therapy" OR TI "self-management" OR AB "self-management") AND (MH "Randomized Controlled Trials+" OR TI randomized OR AB randomized OR TI randomised OR AB randomised OR TI randomly OR AB randomly OR TI placebo OR AB placebo OR TI trial OR AB trial) | Search terms adapted for CINAHL syntax. |
| **CNKI** | Inception to December 2025; bridge search on 20 April 2026 | 主题=(低血糖 OR 血糖过低 OR 低血糖症) AND 主题=(恐惧 OR 害怕 OR 担忧 OR 忧虑 OR 焦虑 OR 心理困扰 OR 心理负担 OR 低血糖恐惧 OR 低血糖恐惧感 OR 低血糖担忧) AND 主题=(2型糖尿病 OR Ⅱ型糖尿病 OR II型糖尿病 OR 成人发病型糖尿病 OR 非胰岛素依赖型糖尿病) AND 主题=(干预 OR 教育 OR 健康教育 OR 护理 OR 护理干预 OR 心理干预 OR 行为干预 OR 认知行为 OR 自我管理 OR 家庭支持 OR 数字化 OR 随访) AND 主题=(随机 OR 随机对照 OR 随机对照试验 OR 对照试验 OR 临床试验) | Chinese database search. |
| **WANFANG** | Inception to December 2025; bridge search on 20 April 2026 | 主题=(低血糖 OR 血糖过低 OR 低血糖症) AND 主题=(恐惧 OR 害怕 OR 担忧 OR 忧虑 OR 焦虑 OR 心理困扰 OR 心理负担 OR 低血糖恐惧 OR 低血糖恐惧感 OR 低血糖担忧) AND 主题=(2型糖尿病 OR Ⅱ型糖尿病 OR II型糖尿病 OR 成人发病型糖尿病 OR 非胰岛素依赖型糖尿病) AND 主题=(干预 OR 教育 OR 健康教育 OR 护理 OR 护理干预 OR 心理干预 OR 行为干预 OR 认知行为 OR 自我管理 OR 家庭支持 OR 数字化 OR 随访) AND 主题=(随机 OR 随机对照 OR 随机对照试验 OR 对照试验 OR 临床试验) | Chinese database search. |

Note: No restrictions were applied by publication year because searches covered the period from database inception to December 2025. Peer-reviewed articles published in English or Chinese were eligible for inclusion. Search terms and indexing terms were adapted to the syntax of each database. Randomised controlled trial terms were included because only RCTs were eligible for inclusion.
