# Supplementary material for: Educational and behavioural interventions for reducing fear of hypoglycaemia in adults with type 2 diabetes: A systematic review and meta-analysis of randomised controlled trials
Source: PLoS One. 2026 Oct 5;21(10):e0358881. doi: 10.1371/journal.pone.0358881 (PMC13637911; doi:10.1371/journal.pone.0358881)
Supplement: Supplementary file 3 [file pone.0358881.s007.docx]

**S5 Table. TIDieR-style summary of intervention characteristics**

**Note:** This table summarises the intervention characteristics of the included trials using a TIDieR-style format. Information was extracted from the published articles and the prespecified extraction dataset. “Not clearly reported” indicates that the relevant item was not sufficiently described in the extracted source material. FOH, fear of hypoglycaemia; T2DM, type 2 diabetes mellitus; TIDieR, Template for Intervention Description and Replication.

| **Study** | **Rationale / theoretical basis** | **Intervention components / materials** | **Provider and mode of delivery** | **Setting, intensity, and duration** | **Comparator** | **Tailoring, fidelity, and adherence reporting** |
| --- | --- | --- | --- | --- | --- | --- |
| Yan et al. 2024 | King’s Theory of Goal Attainment; online feedback to support blood glucose management and reduce FOH-related behaviour and worry. | Online feedback intervention. Specific materials were not fully described in the extracted dataset. | Provider not clearly reported in the extracted dataset. Delivered through an online feedback format. | Digital/remote support; 24 weeks. Assessment at baseline and 24 weeks. | Comparator described in the source trial; details were not fully specified in the extracted dataset. | Individual feedback was implied by the online feedback approach, but tailoring procedures and intervention fidelity/adherence reporting were not clearly reported. |
| Shen et al. 2022 | Common-Sense Model (CSM); illness perception education for patients with impaired awareness of hypoglycaemia. | CSM-based illness perception education with follow-up. | Provider not clearly reported in the extracted dataset. Educational intervention with follow-up contact. | Clinical/educational support setting; 12 weeks. Assessments at baseline, 1 month, and 3 months. | Usual or standard care comparator as described in the source trial. | Tailoring and fidelity procedures were not clearly reported in the extracted dataset. |
| Sheikhi et al. 2020 | Eye Movement Desensitisation and Reprocessing (EMDR); psychological therapy targeting emotional responses to hypoglycaemia. | Two EMDR sessions, each lasting 45 minutes. | Delivered as psychological therapy; provider training and delivery details should be interpreted according to the source trial. | Two 45-minute sessions; follow-up assessments at 1 and 3 months after intervention. | Control condition as described in the source trial. | Fidelity/adherence details were not fully captured in the extracted dataset. |
| Xu et al. 2023 | Exercise of Self-Care Agency (ESCA) scale combined with Information-Motivation-Behavioural Skills (IMB) model-based targeted nursing intervention. | Targeted nursing intervention informed by ESCA and IMB model principles. | Nursing-led intervention was implied by the intervention description; detailed provider characteristics were not clearly reported. | Inpatient setting; delivered during hospitalisation. Assessments before and after intervention. | Routine or standard nursing/diabetes care as described in the source trial. | Targeting was implied by the model-based nursing approach; fidelity and adherence reporting were not clearly described. |
| Liu et al. 2023 | Hope theory-based education and psychological support for patients with T2DM who had experienced hypoglycaemia. | Hope theory-based education combined with psychological support. | Provider not clearly reported in the extracted dataset; educational and psychological support format. | 4 weeks. Assessments before and after intervention. | Routine or standard care as described in the source trial. | Tailoring, adherence, and fidelity procedures were not clearly reported in the extracted dataset. |
| Liang et al. 2021 | Stress inoculation training to address hypoglycaemia-related worry in young and middle-aged adults with T2DM. | 8-week stress inoculation training programme. | Provider not clearly reported in the extracted dataset; psychological/behavioural training format. | 8 weeks. Assessments before and after intervention. | Routine or standard care as described in the source trial. | Tailoring and fidelity reporting were not clearly described in the extracted dataset. |
| Zhou et al. 2019 | Multidisciplinary in-hospital consultation and education for diabetic patients with hypoglycaemia experience. | Multidisciplinary consultation and education during hospitalisation. | Multidisciplinary team involvement was indicated; detailed provider roles were not clearly reported. | Inpatient setting; intervention delivered during hospitalisation. Follow-up at 6 months. | Routine inpatient care as described in the source trial. | Fidelity and adherence reporting were not clearly described in the extracted dataset. |
| Lin et al. 2017 | Traditional Chinese medicine-based emotional nursing to address emotional responses and FOH in T2DM. | Traditional Chinese emotional nursing during hospitalisation. | Nursing-led intervention was implied by the intervention description; detailed provider characteristics were not clearly reported. | Inpatient setting; delivered during hospitalisation. Assessment at the end of intervention. | Routine nursing or standard care as described in the source trial. | Tailoring, adherence, and fidelity procedures were not clearly reported in the extracted dataset. |
| Zhao et al. 2025 | Behaviour Change Wheel (BCW)-based health education targeting FOH behaviour and worry. | Health education with in-person sessions and online coaching. | Delivered through in-person education and online coaching; detailed provider roles should be interpreted according to the source article. | Inpatient phase plus 3 weeks of online coaching; assessments at T0, T1, and T2. | Control condition as described in the source trial. | Online coaching suggests some ongoing support; detailed tailoring and fidelity/adherence reporting should be interpreted according to the source article. |
| Büyükkaya Besen et al. 2025 | Digital diabetes clinic support for individuals newly initiated on insulin therapy. | Digital diabetes clinic continuous support programme. | Digital clinic support; provider team details should be interpreted according to the source article. | Digital/clinic-supported programme; 12 weeks of active support. Assessments at baseline, 3 months, and 6 months. | Control condition as described in the source trial. | Continuous digital support suggests ongoing contact; detailed tailoring, adherence, and fidelity reporting should be interpreted according to the source article. |
| Rondhianto et al. 2024 | Family Caregiver Empowerment Model targeting FOH in people with T2DM and recent hypoglycaemia. | Family caregiver empowerment intervention involving patient-caregiver dyads. | Provider not clearly reported in the extracted dataset; family-based empowerment format. | 10-week intervention, with post-test 2 weeks after intervention. | Control condition as described in the source trial. | Family-caregiver involvement suggests dyadic support; fidelity/adherence procedures were not fully captured in the extracted dataset. |

**Additional note:** The table is intended to improve transparency of intervention reporting. It should not be interpreted as a full fidelity assessment, because several included studies did not report sufficient details on intervention materials, provider training, tailoring procedures, or adherence monitoring.
